# Location location location: a carbon footprint calculator for transparent travel to the UN Climate Conference 2022

**DOI:** 10.14324/111.444/ucloe.000066

**Published:** 2023-11-29

**Authors:** Jonathan Barnsley, Jhénelle A. Williams, Simon Chin-Yee, Anthony Costello, Mark Maslin, Jacqueline McGlade, Richard Taylor, Matthew Winning, Priti Parikh

**Affiliations:** 1 Department of Geography, North-West Wing, University College London, Gower Street, London, WC1E 6BT, UK; 2 Department of Political Science, The School of Public Policy, University College London, The Rubin Building, 29/31 Tavistock Square, London, WC1H 9QU, UK; 3 Institute for Global Health, Institute of Child Health, University College London, 30 Guilford Street, London, WC1N 1EH, UK; 4 Institute for Global Prosperity, University College London, Maple House, 149 Tottenham Court Road, London, W1T 7NF, UK; 5 UCL Institute for Sustainable Resources, University College London, 14 Upper Woburn Place, London, WC1H 0NN, UK; 6 Bartlett School of Sustainable Construction, 22 Gordon St, London WC1H 0AY, UK

**Keywords:** carbon footprint, climate change, climate justice, COP, carbon offsetting

## Abstract

Addressing the large carbon footprint of conferences such as the United Nations Climate Change Convention Conference of the Parties (COP) will be important for maintaining public confidence in climate policy. Transparency is also a vital aspect of creating equitable outcomes in climate policies, as those most likely to be affected or who can create change on the ground are often unable to attend in person because of the high financial costs as well as having a large carbon footprint. The selection of host locations for the regular meetings of the UN Climate Change Convention is based on a rotation amongst the five UN regions, which for 2022 was Africa. Here, we present a carbon footprint calculator for travel to COP 27 in Sharm El-Sheikh, Egypt, weighing the benefits of certain routes and modes of transport. The calculator demonstrates the well-known carbon efficiency of coach and rail over flights but shows that these benefits were partly diminished in the case of COP 27 due to insufficient transport links from Europe to the conference location. However, we also highlight some of the benefits of hosting a COP in the Global South, particularly in the context of climate justice. Users of the calculator are invited to consider all their options for travel and acknowledge the issue of climate justice through careful selection of carbon offsets.

## Introduction

The transport sector is a major contributor to climate change, accounting for approximately 23% of global energy-related greenhouse gas emissions in 2019 [1]. If global warming is to be limited to 2°C, models project that transport emissions must decrease by 29% of their 2020 amount by 2050 [1]. To limit warming to 1.5°C, a reduction of 69% would be required [1]. In the long-term, potential avenues for decarbonisation include the widespread electrification of vehicles, bio-based fuels or hydrogen to replace fossil fuels [2]. However, the large-scale infrastructure changes required to implement these solutions, and the urgency with which decarbonisation must occur, signify that alternative short-term solutions may also need to be implemented to reach these targets. Short-term mitigation of greenhouse gas emissions can take place through systemic changes that alleviate the demand for transport and incentivise greener alternatives. For example, increased digitalisation of commerce and expanded public transport links could represent short-term policies that reduce greenhouse gas emissions whilst long-term policies such as the electrification of transport wait to take effect [3,4].

Although governments ultimately hold the keys to systemic change through levers such as subsidies and taxation, public engagement is an important factor in influencing government policy. However, public engagement relies on access to accurate information that then informs personal choice. In the context of climate change, this information is primarily conveyed through the vehicle of a carbon footprint. Since its emergence in the early 2000s, the carbon footprint has increasingly been used to measure the impact an individual, business or institution has on the climate [5]. Distilling these effects down to a single number requires a level of abstraction that can obfuscate some of the subtleties of climate change. For example, two carbon footprints might compare differently to each other were they to be judged by their climate impact on a 20-year horizon rather than a 100-year horizon [6]. Furthermore, the de facto unit for carbon footprints, tonnes of carbon dioxide equivalent (tCO_2_e), is difficult to gauge in an absolute sense without comparison to another footprint. Nonetheless, the carbon footprint has become an essential metric, not only for individual choice, but for domestic policy and international negotiation.

Since the development of carbon markets in 2005, it has been possible to associate each carbon footprint with a monetary cost. This provides the means for individuals to gauge the value of their personal choices with regards to climate change and to make informed decisions based on that valuation. It has also become commonplace for individuals and organisations to ‘offset’ their carbon footprint by purchasing carbon credits, and services facilitating this are increasingly abundant. However, these services often lack transparency in their measurement of the carbon footprint and the schemes through which the carbon will be offset [7].

Fundamental to the decarbonisation effort is the Conference of the Parties (COP), at which member States party to the United Nations Framework Convention on Climate Change (UNFCCC) meet annually to discuss and negotiate climate policy. However, global summits such as COP inevitably have a climate impact of their own due to the energy costs of travel, accommodation, food, water and waste. In 2021, COP 26 was criticised in the press for posting the largest carbon footprint of any COP to date [8]. The average delegate at COP 26 produced a footprint of 3.42 tCO_2_e, comparable in size to the average annual footprint of individuals in countries such as India, Brazil or Egypt [9,10]. The overall carbon footprint of the conference was comparable to the annual emissions of a small island nation such as Samoa [9,10]. Going forward, the conference must be transparent about its large carbon footprint and address the elephant in the room: Why, in the post-pandemic era, where online conferencing has become normalised, does the physical conference of COP need to be so large? Failure to address the public’s concern could undermine confidence in climate policy, increasing resistance to the progressive domestic policy that COP aims to promote. There is therefore an ever greater imperative to measure, minimise and offset the carbon footprint of a COP to ensure that those attending the conference, in working to combat climate change, do not inadvertently exacerbate it.

### Carbon footprint calculators

There currently exists a wealth of online carbon footprint calculators developed by governments, non-profits, charities or private companies [11]. For those booking travel abroad, they are likely to encounter carbon footprint calculators at various stages. Flight searching tools, airline booking websites, rail operators and even Google Maps often will provide some information about a user’s carbon footprint. Some of these services will also provide the option to ‘offset’ their footprint for a small fee. These calculators vary in their methodology and transparency. For example, the Google Flights calculator is based on the European Environment Agency model [12], which accounts for many aspects of flight, including the aircraft, altitude and passenger density. However, it is not standard for calculators to reference their methodology, and many do not. The transparency in carbon offsetting is similarly mixed.

One challenge for visitors to COP is to build a cohesive picture of how the carbon footprints of various modes of transport compare against one another. Particularly in the case of international train travel, national rail networks can vary significantly in terms of their energy use and passenger density. The UK rail operator LNER uses a simple model with a linear relationship between distance and emissions, but the gradient of that linear model would be different in other countries with different energy and rail infrastructure. This places an unreasonable amount of work on consumers to receive carbon footprint quotes from multiple rail companies, airlines and possibly estimates for coach or car before they can make a fully informed decision about their choice of transport.

Here we present a carbon footprint calculator for travel between the UK and COP 27 in Sharm El-Sheikh. By developing our own carbon footprint calculator, we hope to achieve three main goals: Firstly, we aim to increase public awareness of the importance of the choice of transport using a side-by-side comparison of direct flights with less carbon intensive modes of transportation, including a discussion about the possible indirect effects of aviation. Secondly, we call for increased transparency in carbon footprint calculators and set a precedent by ensuring all data and calculations are open source. Thirdly, we highlight the option of attending virtually by comparing the footprint of online conferencing against in-person travel. This is made explicit in the user interface of the calculator, which asks users to seriously consider whether their physical attendance at the conference is necessary before allowing them to continue. Where it is necessary, users are directed towards carbon offsetting schemes that support the UN’s Sustainable Development Goals (SDGs). This approach intends to upend the idea of carbon offsets as just a carte blanche for emissions, reprioritising emissions reduction and instead harnessing carbon offsets as a lever for sustainable development. Ultimately, we aim for users to come away more informed about the choices that they make when travelling internationally, the limitations of carbon footprint measurement, and the best practices for carbon offsetting. The full calculator can be accessed through the UCL climate hub at https://www.ucl.ac.uk/climate-change/cop27-carbon-footprint-calculator.

### Methodology and data

**Figure 1:**
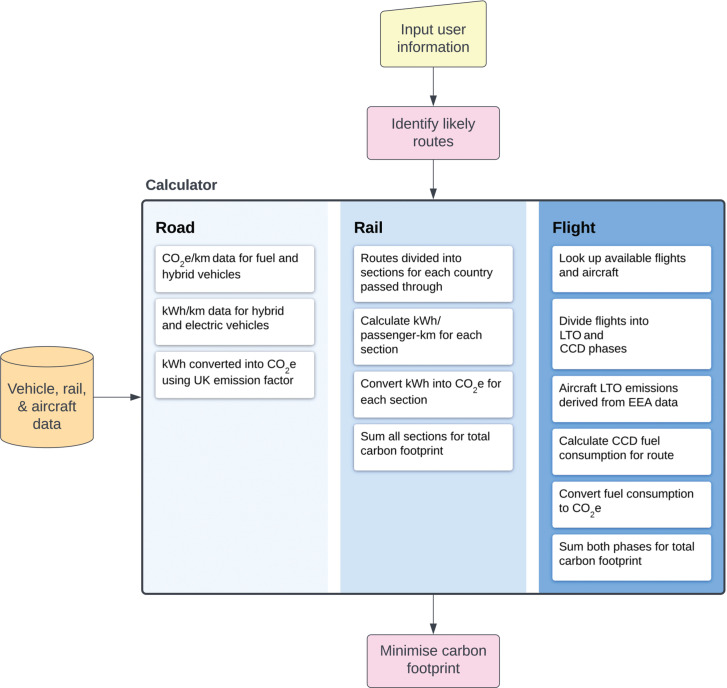
Flow chart representing the carbon footprint calculator, flowing from top to bottom. Model components are represented in shades of blue (coach and passenger car both represented by ‘Road’), with important features listed.

The calculator presented here draws inspiration from existing models created by the European Environment Agency [13], the International Civil Aviation Organisation [14] and the UK Department for Business, Energy, and Industrial Strategy (BEIS) [15]. Some elements have been simplified for the purpose of this project. However, the use-case of this model – for individuals and not organisations – has at times allowed a more detailed approach. The model consists of four main components that represent each mode of transport: aircraft, rail, car and coach (Fig. 1). The default starting point of the model is London but can be changed by the user to one of seven cities distributed across the UK. Fifteen further cities form a network of travel links, any combination of which may be compiled to form a route from the UK to Sharm El-Sheikh. However, the typical user will be presented with four possible routes that have been chosen to represent varying degrees of convenience versus carbon efficiency. Routes that traverse areas classified by the UK foreign office as ‘Advise against all travel’, such as Iraq and Libya, have been excluded. Many possible ferry routes have also been discounted as these services have been discontinued since the Covid-19 pandemic. The combined result of this is that no reasonable safe route exists between the UK and Sharm El-Sheikh by land or sea and all routes must fly over the Mediterranean Sea. The carbon benefits of travelling part-way by land are explored by comparing a direct flight with three routes that utilise rail or road up to a waypoint – either Brussels, Milan or Istanbul – before flying into Sharm El-Sheikh. These specific waypoints are chosen for their accessibility by rail and the existence of a direct flight to Sharm El-Sheikh from their respective airports. The output of the model presents each route alongside its carbon footprint and travel time. Although the output of the model is specific to COP 27, some components of the model are scalable in such a way that they can be repurposed for future COPs, other international conferences or even any two global cities. However, the detailed aircraft data that feeds into the flight component has necessitated a narrower approach within the scope of this project.

International flights were identified through searches on Google Flights and SkyScanner. Cars and coaches utilise road distance data, sourced through the Google Maps API, to generate distances between city nodes in the model. This distance is then also adopted by the rail component, based on the assumption that the paths of motorways and railways are generally determined by the same features, that is, population centres and topography. Once distances have been established, each model component considers the information relevant to that mode of transport to generate an associated carbon footprint.

The methodology for each component is described here as one of three tiers, which coincide with definitions in the European Environment Agency’s emission inventory guidebook [12]. Tier 1 approaches assume a simple linear relationship between activity data and emission factors. They assume typical or average conditions and are largely independent of technology. Tier 2 approaches are similar but apply country-specific data where conditions may vary by location. Tier 3 approaches employ more sophisticated, physically based models.

### Flights

Flight emissions are based on a tier 3 approach. Greenhouse gas emissions are calculated from fuel consumption using the Intergovernmental Panel on Climate Change (IPCC)’s emission factors for jet fuel and converted into CO_2_e by the global warming potential (GWP) factors outlined in the IPCC’s sixth assessment report [16]. The calculation of fuel consumption is divided into the landing/take-off (LTO) and climb, cruise, descent (CCD) phases of flight. Data on LTO consumption for each aircraft is provided by the EEA’s ‘master calculator’, which assumes the most common engine types for each aircraft model and the average taxi time for European airports in 2015 [13]. Fuel consumption during the CCD phase is modelled by the Breguet range equation for jet-engine aircraft in steady flight [17]:

$$m_{1}=m_{2}e^{\left(\frac{Rgb_{f}}{vL/D}\right)},$$

where:

*m*_1_ – take-off mass (*t*)

*m*_2_ – landing mass (*t*)

*e* – Euler’s number ≈2.72

*R* – range (*m*)

*g* – acceleration due to gravity ≈9.81 ms^−2^

*b_f_* – thrust-specific fuel consumption (kgN^−1^s^1^)

*v* – velocity (ms^−1^)

*L*/*D* – lift/drag ratio

Thus, the model employs a bottom-up approach that interprets the fundamental aerodynamic properties of aircraft to determine fuel efficiency on a flight-by-flight basis. In theory, this approach should give us more confidence in the results than a top-down passenger-km-based approach such as that by the BEIS [15]. However, it remains a highly idealised representation, excluding, for example, fuel consumption for engine processes other than thrust.

Comparison of the model with the European Economic Area (EEA)-equivalent [13] shows that for a typical aircraft, fuel consumption is 17% lower for the shortest flights and 10% higher for the longest flights than the EEA’s estimations, with medium-length flights loosely agreeing. These discrepancies are likely the result of several simplifications in the model. For example, the exclusion of freight, which varies in impact from region to region. The International Civil Aviation Organization (ICAO)’s calculator also draws data from a custom-made tool to approximate aircraft efficiency beyond those numbers reported by Boeing and Airbus [18]. However, this data has not been made public. Nonetheless, our calculator produces comparable results to these more sophisticated models.

Following these aircraft-level calculations, fuel consumption is divided among passengers according to their flight class. Exact seat configurations vary from airline to airline and with the destination of the flight. However, rough seat numbers have been approximated from airline customer-information and third-party websites [19,20]. For simplicity, business and first class have been combined in the model as ‘premium’. These are then compared with the maximum passenger capacity published by Boeing and Airbus to calculate the effective ‘premium multiplier’ – that is, the number of economy seats that would occupy the space taken by each premium seat (Table 1). Per-passenger fuel consumption is finally converted into GWP using the IPCC conversion factors and forms the output of this model component.

**Table 1.** The max seats, common seating configurations and associated premium multiplier for a selection of aircraft [20–22]

Name	Max seats	Premium seats	Economy seats	Total seats	Premium multiplier
Airbus A320	180	12	138	150	3.5
Boeing 777-300	550	70	229	299	4.6
Boeing 787-9	290	54	192	246	1.8

### Passenger cars

The emissions calculator for passenger cars employs a tier 1 approach but utilises vehicle-specific efficiency data reported to the EEA under EU regulation 2019/631. As per regulation, these efficiency measurements reflect a driving pattern defined by the Worldwide Harmonised Light Vehicle Test Procedure (WLTP), which aims to recreate a variety of typical driving conditions from suburban to open road. Fuel vehicles are allocated a direct footprint in kgCO_2_e/km. For hybrid and electric vehicles (EVs), electricity consumption is converted using the UK greenhouse gas conversion factor of 0.19338 kgCO_2_e/kWh [15].

As trans-continental journeys are certain to charge or refuel their vehicles in countries other than the UK, the approximation that all electricity is converted using UK factors is a notable limitation of the model, most impactful for EVs. However, uncertainties in the initial battery charge, battery degradation and driver behaviour represent a significant modelling challenge when estimating charging locations. An example journey through the UK, France and Italy would very likely be overestimated by the model due to France’s significantly lower dependency on fossil fuels for electricity (Table 2). Furthermore, WLTP driving conditions do not necessarily reflect all journey types, particularly long motorway-dominated routes that are common for international travel. For a more comprehensive approach, a vehicle activity-based model such as the EEA’s COPERT could be employed to include these effects [24].

**Table 2.** Greenhouse gas conversion factors for electricity generation in the UK, France and Italy in 2019 [23]

Country	kgCO_2_e/kWh
UK	0.225
France	0.063
Italy	0.234

UK figures have since been reported independently of EU data.

Car journeys across the English Channel assume use of the Eurotunnel from Folkestone to Calais, as this is estimated to be an order of magnitude more efficient than the ferry alternatives [25]. This does not employ the same footprinting methodology as the train component due to fundamental differences in the vehicle design and passenger density. However, Eurotunnel report the carbon footprint of the crossing to be 2 kgCO_2_e/car [25].

### Rail

Railways pose several unique challenges in establishing a carbon footprint. For example, whereas the passenger load factor of flights stays relatively constant throughout time, the passenger load of a train varies widely according to the ebb and flow of commuter patterns. Unlike aircraft, the carbon efficiency of a train depends not only on its fundamental engineering, but also the number of stops en route, the number of carriages it hauls, and each of these vary by route and throughout any given day. It is safe to assert that electric trains are lower emitters of greenhouse gases than diesel trains, but the exact extent is tied to the fossil-fuel dependency of the electricity on which it operates [24]. The model therefore aims for a tier 2 approach that averages over some of these effects, whilst still reflecting the variability in railway and energy infrastructure across Europe.

Due to disruption by the Covid-19 pandemic in 2020, 2019 is used as the closest analogue to 2022 in terms of rail travel statistics. Passenger and energy data is combined with electricity conversion factors to produce a kgCO_2_e/passenger-km figure for each EU member state (Table 3). Journeys by rail in the model are then calculated according to the country in which they take place, or mostly take place in the case of international travel.

**Table 3.** Railway passenger and energy data for a selection of countries in the model and their consequent carbon footprint per passenger-km [26]

Country	Passengers (millions passenger-km)	Electricity consumption (GWh)	Electricity emission factor (kgCO_2_e/kWh)	Carbon footprint (kgCO_2_e/passenger-km)
France	96,500	8470	0.063	0.0069
Italy	56,600	5550	0.234	0.0287
UK	71,800	5090	0.296	0.0262
Austria	13,200	1990	0.078	0.0146
Romania	5910	1000	0.255	0.0541
Bulgaria	1520	311	0.362	0.0927

This approach makes two key assumptions in order to calculate a carbon footprint: Firstly, it assumes that electricity consumption is dominated by passenger rail as opposed to freight. In the UK at least, freight is mostly powered by diesel and accounted for only 1.6% of railway electricity consumption in 2019–20 [27]. However, similar statistics for every EU member state were not available to incorporate into this methodology. Secondly, the approach must contend with the issue that not all passenger-km take place on electrified railway lines. Approximately 60% of the European rail network is electrified, but as the busiest lines have been prioritised for electrification, 80% of rail traffic is covered by this amount [28]. The passenger-km in Table 3 are therefore first scaled by 0.8 to account for this effect. However, more granular data on electrified passenger-km for each member-state would be preferred.

### Coach

The remaining coach component of the model employs a simple tier 1 approach to calculating emissions, utilising data from the UK database of greenhouse gas conversion factors [15]. The model does not discern between different types of coach, but there is an assumption that variability in efficiency is less than in cars, where models stretch from ultra-efficient EVs to high-performance sports cars. As coaches generally have diesel engines, they avoid the complications related to electricity consumption and therefore require only a simple calculation to provide a carbon footprint. The model utilises the same road distance data as that in the car component and multiplies by the per-km BEIS conversion factor to arrive at a carbon footprint.

Coach journeys that cross the English Channel also assume use of the Eurotunnel, as in the car component. Unlike the car, however, Eurotunnel have not reported a carbon footprint per vehicle for coaches. The model instead approximates a coach as similar in size to a freight vehicle, for which Eurotunnel report a carbon footprint of 9 kgCO_2_e/vehicle [25]. As the vehicle engines are turned off during travel, the size of a vehicle is the leading factor in its carbon footprint on the Eurotunnel. This would imply that a coach was approximately equivalent to 4.5 cars, passing a basic sanity check. The calculator uses a figure of 0.2 kgCO_2_e/person, which would be equivalent to 9 kgCO_2_e divided amongst 45 passengers (Flixbus coaches, for example, seat up to 65 passengers between London and Paris). Although there are large uncertainties in this estimate, their effect on the journey’s total footprint are negligible, as any footprint less than 1 kgCO_2_e will be dwarfed by the remaining journey to Sharm El-Sheikh.

### Digital delegation

In addition to carbon footprints for a selection of routes between the UK and Sharm El-Sheikh, users are also provided with a carbon footprint associated with ‘Digital delegation’, which involves accessing the conference virtually through the use of online conferencing software. COP 26 provided its own platform for participants to engage with the conference both synchronously in the form of livestreamed presentations and asynchronously through written summaries of negotiations or recorded material [29]. A similar platform had not been announced for COP 27, although repurposing the platform from the previous year would be a simple and effective solution. Digital delegation allows for the inclusion of many more people than would be possible with a purely physical conference. However, we accept that even this form of accessibility will not be available to everyone, given the limitations in access to broadband and computer hardware. Furthermore, an online platform requires the consideration of time zones when planning important events at COP, ensuring that events concerning a particular global region take place at a time when digital delegates from that region can reasonably attend.

Energy consumption associated with digital delegation can be split into two categories: energy consumed locally by the computer for essential processes (e.g., lighting the screen) and energy consumed by the network for transmitting data from the user to the conference and vice versa. As computers are often also used during in-person meetings for note taking or presenting, we discount local energy consumption here and focus entirely on the carbon footprint of data transfer. Aslan et al. estimate the power consumption of data transfer in 2015 to be 0.06 kWh/GB [30]. However, they also note a decreasing trend in power consumption associated with increased network efficiency over time. Since 2000, the power consumption of data transfer has halved approximately every two years. Extrapolating this trend to 2022, we arrive at a power consumption of 0.0053 kWh/GB. Here, we use published figures by Microsoft to approximate the data requirements of live streaming [31]. Assuming a generous conferencing time of 8 hours/day, 5 days/week, we estimate a total power consumption of 1.24 kWh/user. The BEIS Strategy estimates the carbon footprint of UK electricity to be 0.19338 kgCO_2_e/kWh [15]. This results in an overall carbon footprint of digital delegation of 0.24 kgCO_2_e.

## Results and discussion

**Figure 2:**
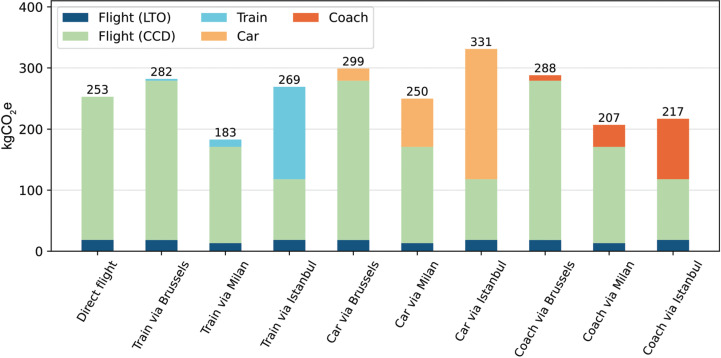
A selection of journeys between London and Sharm El-Sheikh and their associated carbon footprints, coloured by mode of transport. Includes emissions associated with the LTO and CCD phases of flight. Routes ‘via’ a waypoint travel by the specified land transport up to the waypoint before flying the remainder of the route to Sharm El-Sheikh. All flights are economy seats, and all car journeys are calculated for a diesel Ford Fiesta with two passengers.

Model results for a selection of journeys between London and Sharm El-Sheikh (SHS) show a range of possible carbon footprints between 183 and 331 kgCO_2_e (Fig. 2). A direct flight is associated with a carbon footprint of 253 kgCO_2_e, approximately one thousand times the footprint of digital delegation. Outside of digital delegation, travelling part of the way by coach or train offers the greatest gains in efficiency. However, there are some notable exceptions to this pattern. The model finds that flying from Brussels is not beneficial in any scenario. Despite the 300 km shortening of the flight relative to London–Sharm El-Sheikh, the less efficient aircraft – in this case a Boeing 737-800 versus the more efficient Airbus A320 – results in a larger overall carbon footprint. Similarly, the model finds no advantage in routing by train via Istanbul. When compared with a route via Milan, flying from Istanbul cuts flight emissions by 31%, but increases rail emissions by 1250%. The underlying differences behind this are related to electricity emissions and rail efficiency along each route. Whereas the Milan route utilises rail mostly in France, where electricity is very carbon cheap, the Istanbul route spends considerable distance in Bulgaria, which has one of the highest emission factors per kWh in Europe. Romania and Bulgaria also carry far fewer passengers per kWh spent, compounding this effect. This would normally be offset by savings in flight emissions. However, the inclusion of LTO emissions in the model demonstrates that this is not as effective as simpler models might suggest. LTO accounts for 15% of total emissions on a flight from Istanbul to Sharm El-Sheikh. Although the flight from Istanbul is roughly half the distance of a flight from Milan, it is only a third less emitting.

Nonetheless, the differences between routes are relatively small in the context of the larger carbon footprint. The greatest carbon saving available is to travel by train via Milan, a reduction of only 40% relative to the direct-flight option. These carbon reductions come at a significant time and financial cost, which make it unlikely to be a viable option for travel to Sharm El-Sheikh this November. These results are partly a consequence of the necessary flight across the Mediterranean and the LTO and CCD model for flight carbon. Even in routes with significant mileage by land transport, the flight emissions dominate the overall carbon footprint. The LTO emissions represent a minimum footprint of each flight, which is approached as flight distance is decreased. Shorter flights therefore make smaller efficiency gains from cutting flight distance than longer flights.

### Comparison with COP 26

**Figure 3:**
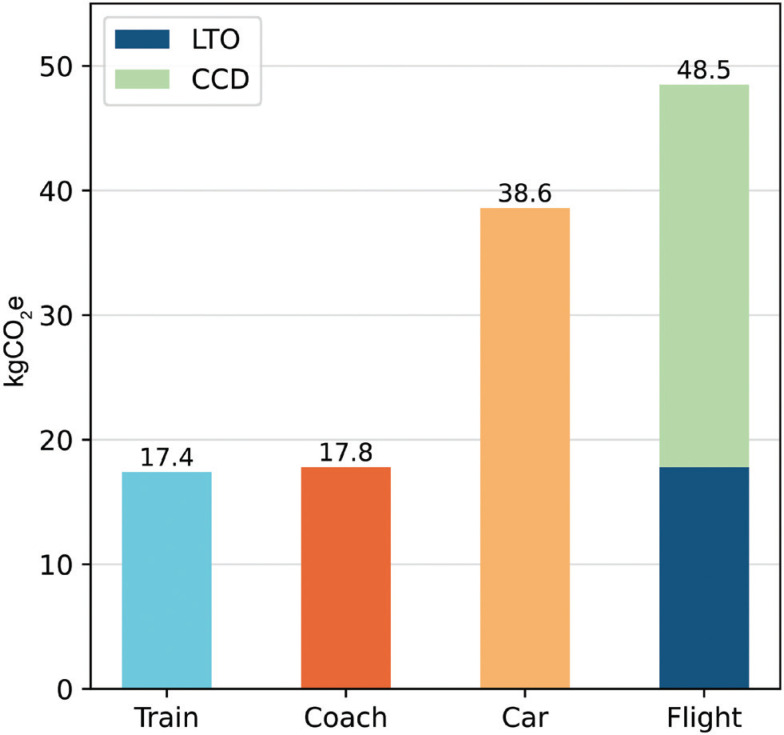
The carbon footprints associated with various modes of transport between London and Glasgow. Includes emissions associated with the LTO and CCD phases of flight.

To assess the model in a situation where flights are not a necessity, equivalent results are presented for COP 26 in Glasgow, had the calculator been available that year (Fig. 3). The model once again shows increased carbon efficiency by rail and coach, up to 64% lower emissions than the direct flight.

However, unlike the Sharm El-Sheikh variant, these carbon savings do not come at such expense in time and money. In fact, factoring in an early arrival time at the airport, flying from London to Glasgow is at most a one-hour time saving on the rail alternative. This comparison motivates the definition of a carbon time efficiency for a given route, measured in kgCO_2_e saved per hour spent travelling above the direct flight baseline:

$$\text{Efficiency=}\frac{{\text{flight CO}}_{2}\text{e}-{\text{route CO}}_{2}\text{e}}{\text{route time}-\text{flight time}},$$

where flight CO_2_e and flight time are the carbon footprint and length in hours of the direct flight respectively, and route CO_2_e and route time are the total carbon footprint and length in hours of an alternate route that utilises more carbon efficient modes of transport for at least part of the journey. This value is a metric for how practical a flight alternative is for time sensitive travel. Low-emission routes are scaled by their time efficiency relative to the faster (but higher-emitting) flight alternative. Under this metric, COP 26 and COP 27 can be directly compared for their carbon time efficiency. It also allows individuals to set a ‘value’ on their own time in terms of carbon – a threshold past which they would be willing to sacrifice some time in return for lower emission travel.

**Figure 4:**
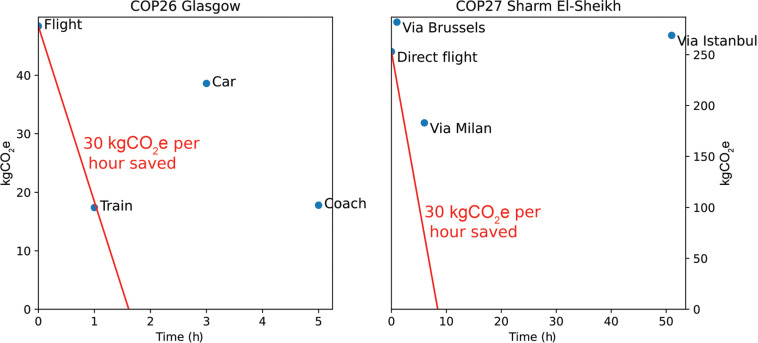
The carbon footprint of various routes to (left) Glasgow and (right) Sharm El-Sheikh from London, plotted against the length of the journey in hours. The red line indicates points equal to a 30 kgCO_2_e/h saving from the direct flight option in each case. The COP 27 routes utilise rail transport up to the specified city, then fly direct to Sharm El-Sheikh.

For example, taking the train to Glasgow instead of flying represents a saving of approximately 31 kgCO_2_e/h. Whether this is a satisfactory trade-off is a subjective opinion. It depends on how an individual values their time and how committed they are to minimising their carbon footprint. However, for the purpose of comparison, we consider a typical climate-conscious visitor to COP 26 who sees this as a worthwhile trade-off and would opt to take the train. Is there then an equally efficient route to COP 27, which saves carbon at a comparable or cheaper time cost? Figure 4 explores this by plotting time against carbon footprint for a selection of journeys to both COP 26 and COP 27. A threshold of 30 kgCO_2_e/h is drawn in red. Any journeys beneath and to the left of this line are considered worthwhile by the hypothetical individual outlined above. From this, it is clear that no such route exists to COP 27. Fewer UK visitors to COP 27 are therefore likely to choose carbon efficient modes of transport, even if they would have done so for COP 26. These calculations also ignore the financial cost of different modes of travel. The overall ‘cost’ of a journey could be considered as a sum of three costs associated with time, money and carbon. This framework could then be used to assess the most practically responsible methods of transport in any scenario. However, care would have to be taken over the valuation of carbon mitigation versus carbon offsetting – uncertainties in the latter mean that these should not be considered equal.

### Indirect effects of aviation

The carbon footprint calculator only considers direct greenhouse gas emissions when calculating the carbon footprint of flight. However, indirect effects of aviation may also have a significant impact on radiative forcing. As our results would be highly sensitive to how the calculator approaches these effects, we feel it is responsible to discuss them here and justify their exclusion from the calculator. The 1999 IPCC special report on aviation identified three notable indirect effects relating to nitrogen oxide (NOx) emissions, contrail cirrus and aerosols [32]. NOx emissions released from jet fuel combustion have a dual effect of promoting ozone formation whilst depleting methane concentrations [33]. This radiative forcing effect has recently been refined to include a knock-on effect whereby low methane concentrations lead to reduced stratospheric water vapour and a small long-term depletion of ozone that partly offsets the initial increase. Studies completed since the 1999 IPCC special report have worked to quantify this effect, estimating the net effect of NOx emissions to be over half that of CO_2_ [34].

**Figure 5:**
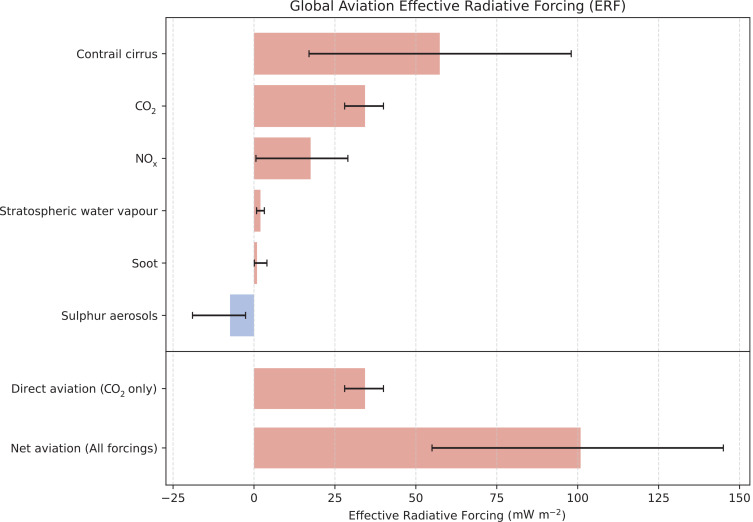
Best estimates of effective radiative forcing (ERF) of aviation between 1940 and 2018 (adapted from [26]). Red bars indicate a warming effect, blue bars indicate a cooling effect. Whiskers indicate the 5–95% confidence interval.

Plane contrails form behind aircraft when the atmosphere is supersaturated with ice [32]. Formed in a straight line, they gradually dissipate into cirrus clouds with a radiative forcing effect nine times greater than the initial contrail [35]. However, there is high uncertainty in both the radiative forcing of cirrus and the distribution of ice saturation in the upper troposphere [36,37]. Amongst other uncertainties, they combine to make the overall contrail effect extremely difficult to estimate [34]. The 5–95% confidence interval puts the radiative forcing of contrail cirrus trails between roughly 50% and 300% of aircraft CO_2_ emissions [34]. Carbon footprint calculators should be cautious about including such highly uncertain effects, as they have the potential to undermine confidence in the calculator itself. However, even the lower-bound estimate of net forcing is 50% greater than CO_2_ effects alone (Fig. 5). Each carbon footprint calculator may approach this differently, but consensus is moving towards approximating net forcing as double the isolated CO_2_ effects [38]. In this case, we have chosen to exclude them from the main calculator, but recommend users at least double the amount of carbon offsets they purchase to account for indirect effects of aviation. Visitors to COP 27 who wish to account for the maximum estimate of indirect effects should multiply the carbon footprint of their flight by 4.5.

### A brief history of carbon-neutral COPs

For those travelling to COP, it is important to know what kind of carbon footprinting and carbon offsetting has already taken place the host government. At the time of publication, this information is not yet available, but previous COPs can give an indication of what might be expected from COP 27. The host country agreement for COP stipulates that the host is responsible for measuring and minimising the carbon footprint of the conference, but the interpretation of how this is achieved and the decision on whether to offset it is left up to the host [39]. Mixed approaches to measuring, minimising and offsetting successive COP footprints have been used. For example, COP 21 in Paris included international travel of UN-accredited visitors in its measurement but excluded it in its offsetting [39]; it was subsequently included in offsetting for COPs 21–25, then COP 26 in Glasgow expanded upon it further to include international travel of accredited visitors and the indirect effects of aviation, which multiplied the carbon footprint of flights by a ‘radiative forcing’ factor of 3 [40]. Offsetting initiatives have also varied significantly: COP 24 in Katowice offset its entire carbon footprint through afforestation projects in Poland [41]; COP 25 in Madrid bought Certified Emission Reductions (CERs) through the EU Emissions Trading Scheme [42]; and COP 26 narrowed this to purchasing mostly CERs or voluntary emission reductions (VERs) with co-benefits defined by the UN’s SDGs.

The expanded measurement of international flights for COP 26 resulted in a significantly higher carbon footprint than previous COPs, over 150% greater than COP 25 in Madrid [10]. Of that footprint, 75% was attributed solely to international flights [40]. COP 26 made clear improvements on previous COPs both in its measurement and offsetting, but it remains to be seen whether this will become the norm for future conferences. Visitors to COP should be conscious of these concerns when planning their travel and any offsetting of their carbon footprint.

### COP, carbon accounting and climate justice

Climate justice is defined by the imbalance between countries that are major contributors to climate change and countries which are most affected by it [43]. It recognises that low-income countries – broadly speaking in the Global South – bear the least responsibility for climate change and yet are the most vulnerable to its effects [44]. COP 27 in Sharm El-Sheikh was the first COP to be hosted outside of Europe since COP 22 in Marrakech. In 2016, Morocco used the opportunity as host nation to shine a spotlight on increasing water scarcity. In doing so, they were able to guide discourse towards one of the key issues that define the climate justice movement [44]. However, the four subsequent European COPs provided less focus on climate justice, failing to secure a commitment to provide finance for loss and damage at COP 26 [45]. In fact, hosting COP in Europe made the conference less accessible to delegates from the Global South by increasing the cost of travel and accommodation. In 2021, the ‘Human Hotel’, a homestay network organised by the COP26 Coalition and Stop Climate Chaos Scotland, arranged local accommodation for 1696 delegates at risk of being priced out of the conference, including scientists, policy makers and indigenous people [46]. Without affordable accommodation, the member states most interested in pursuing climate justice are likely to become the most marginalised by prohibitive costs.

Carbon accounting refers to the legal and financial frameworks that exist to measure and offset carbon emissions. Carbon markets such as the EU Emissions Trading Scheme are a key component of the carbon accounting framework and have been the product of negotiations over many previous COPs. However, the marketisation of carbon fails to address the issue of climate justice. A system for valuing climate action should consider climate change multilaterally in the context of health, infrastructure, food and water security, energy and environment [43]. This could ultimately lead to a new vehicle for individuals to engage with climate change – one which can facilitate the necessary reductions in transport emissions whilst simultaneously supporting sustainable development. In the meantime, users of the carbon footprint calculator are encouraged – after ruling out digital delegation and minimising their footprint – to purchase carbon offsets with co-benefits aligned with the UN’s SDGs.

## Conclusions

Decreasing transport emissions is an important feature of any low-emission pathway. Doing so will require rethinking not only the fuel we use, but how and when we travel at all. Here, we have highlighted some of the ways in which COP can be influential in the discourse around travel. Through the development of UCL’s own carbon footprint calculator, we demonstrate the clear benefits of rail and coach over flights, particularly in the assessment of COP 26. However, the conflicts in Iraq and Libya and the unavailability of trans-Mediterranean ferries necessitated the use of flight for visitors travelling from Europe to COP 27. In these cases, the moderate benefits of travelling part way by rail or coach were offset by the significant time and financial investment of such journeys. The UNFCCC should therefore consider the availability of non-flight transport links when choosing the location of future COPs. Whilst hosting COP outside of Europe is important for promoting equity between member states, the location of Sharm El-Sheikh between conflict-torn countries makes reducing its significant carbon footprint near impossible outside of choosing to use digital delegation.

Those planning to attend future COPs should be aware of several relevant issues before travelling. Firstly, we recommend careful consideration of the necessity of travel and the option of participating virtually. Secondly, it is important to be aware of the carbon footprinting and offsetting already undertaken by governments in advance of COPs. Thirdly, we recommend accommodating for the possible indirect effects of flight by at least doubling the measured carbon footprint, up to a multiplication by 4.5 for maximum effect. Lastly, we encourage conscious engagement with carbon offsetting, opting to purchase carbon credits from projects that support sustainable development and promote climate justice. As in previous years, we expect the carbon footprint of future COPs to receive attention in the media. It is imperative that both the hosts and delegates address this issue transparently to ensure that the organisation of COP is consistent with its decarbonisation messaging.

Future work on the carbon footprint calculator could expand its application to find the most carbon-efficient route between any two locations, not simply between the UK and Sharm El-Sheikh. The calculator could also consider the time and financial cost of each journey. However, great care would need to be taken when valuing carbon mitigation versus carbon offsetting. Lastly, the calculator could address the issue of historical emissions and potentially incorporate this into its recommended offsetting.

## Data Availability

The datasets generated during and/or analysed during the current study are available in the repository: https://www.ucl.ac.uk/climate-change/cop27-carbon-footprint-calculator.
